# Monitoring the interactions between alpha-synuclein and Tau in vitro and in vivo using bimolecular fluorescence complementation

**DOI:** 10.1038/s41598-022-06846-9

**Published:** 2022-02-22

**Authors:** Laura Torres-Garcia, Joana M. P. Domingues, Edoardo Brandi, Caroline Haikal, Janitha M. Mudannayake, Inês C. Brás, Ellen Gerhardt, Wen Li, Alexander Svanbergsson, Tiago F. Outeiro, Gunnar K. Gouras, Jia-Yi Li

**Affiliations:** 1 Experimental Dementia Research Unit, Department of Experimental Medical Science, Lund University, Lund, Sweden; 2 Neural Plasticity and Repair Unit, Department of Experimental Medical Science, Lund University, Lund, Sweden; 3 Developmental and Regenerative Neurobiology, Department of Experimental Medical Science, and Lund Stem Cell Center, Lund University, Lund, Sweden; 4 Department of Experimental Neurodegeneration, Center for Biostructural Imaging of Neurodegeneration, Center for Nanoscale Microscopy and Molecular Physiology of the Brain, University Medical Center Göttingen, Göttingen, Germany; 5 Max Planck Institute for Experimental Medicine, Göttingen, Germany; 6 Faculty of Medical Sciences, Translational and Clinical Research Institute, Newcastle University, Newcastle upon Tyne, UK; 7 Scientific Employee With an Honorary Contract at German Center for Neurodegenerative Diseases (DZNE), Göttingen, Germany; 8 Institute of Health Sciences, China Medical University, Shenyang, China; 9 Present Address: Department of Clinical Neurosciences, University of Cambridge, The Clifford Albbutt Building, Cambridge, UK

**Keywords:** Neuroscience, Alzheimer's disease, Neurodegeneration, Parkinson's disease

## Abstract

Parkinson’s disease (PD) and Alzheimer’s disease (AD) are characterized by pathological accumulation and aggregation of different amyloidogenic proteins, α-synuclein (aSyn) in PD, and amyloid-β (Aβ) and Tau in AD. Strikingly, few PD and AD patients’ brains exhibit pure pathology with most cases presenting mixed types of protein deposits in the brain. Bimolecular fluorescence complementation (BiFC) is a technique based on the complementation of two halves of a fluorescent protein, which allows direct visualization of protein–protein interactions. In the present study, we assessed the ability of aSyn and Tau to interact with each other. For in vitro evaluation, HEK293 and human neuroblastoma cells were used, while in vivo studies were performed by AAV6 injection in the substantia nigra *pars compacta* (SNpc) of mice and rats. We observed that the co-expression of aSyn and Tau led to the emergence of fluorescence, reflecting the interaction of the proteins in cell lines, as well as in mouse and rat SNpc. Thus, our data indicates that aSyn and Tau are able to interact with each other in a biologically relevant context, and that the BiFC assay is an effective tool for studying aSyn-Tau interactions in vitro and in different rodent models in vivo.

## Introduction

Parkinson’s disease (PD) is the most common movement disorder and is characterized by the pathological accumulation of α-synuclein (aSyn) in the form of Lewy bodies (LB) and Lewy neurites (LN), and by the death of dopaminergic neurons in the substantia nigra in the midbrain^1^. Alzheimer’s disease (AD) is the major form of dementia, and the accumulation of amyloid-β (Aβ) and Tau in Aβ plaques and neurofibrillary tangles (NFT), respectively, are pathological hallmarks of AD; however, Tau pathology correlates better with the cognitive decline associated with AD^2^.

Genome-wide association studies (GWAS) have identified the MAPT gene, which encodes for Tau protein; together with the SNCA gene, which encodes for aSyn protein, as major risk factors for the development of PD^3,4^. Furthermore, in human brains co-occurrence of aSyn and Tau pathologies has been described. About 30% of PD postmortem brains show NFTs^5,6^, while LBs and LNs are detected in 60% of cases with AD^7,8^. Moreover, aSyn aggregates are also found in Frontotemporal dementia (FTD)^9,10^, Progressive supranuclear palsy (PSP)^11^ and Down’s syndrome with AD^12,13^. Likewise, Tau accumulation has been observed in Dementia with Lewy bodies (DLB), also called Diffuse Lewy body disease (DLBD)^14–16^; and Parkinson’s disease dementia (PDD)^17,18^. In particular, the co-occurrence of aSyn and Tau in amygdala has been reported in AD, PD, Down’s syndrome and DLB ^14,15,19,20^.

Animal studies have shown that although Tau does not seem to be involved in aSyn spreading in the brain^21^, it plays an important role in synuclein pathology. In mice expressing human PD mutant A53T aSyn, Tau is required for the deficits in learning, memory and synaptic plasticity. A53T aSyn appears to induce Tau phosphorylation by glycogen synthase kinase-3 β (GSK3β) activation, which drives the missorting of Tau to dendritic compartments and an increase in the internalization of AMPA receptors (AMPARs), in the absence of clear neuropathology^22,23^. Additionally, rats virally expressing human Tau (WT or P301L mutant) in substantia nigra *pars compacta* (SNpc) show vulnerability of dopaminergic neurons to Tau-induced neurodegeneration, and human Tau expression leads to a stronger amphetamine-induced rotational behavior than the expression of human aSyn (A30P or A53T mutant) alone in the same area^24^. Similar observations have been described in a mouse model carrying the human K369I Tau mutation, in which impairment in the anterograde axonal transport induced by Tau results in loss of dopaminergic neurons and a Parkinsonian-like phenotype^25^.

Moreover, aSyn appears to be essential for the phosphorylation of Tau in primary neurons after treatment with MPTP, a potent inducer of parkinsonism^1^. Tau co-localizes and interacts with aSyn in aSyn aggregates^26^ and both are found together by immunofluorescence microscopy in the axons of cultured primary hippocampal neurons^27^, and in excitatory pre-synaptic terminals^28^. Furthermore, cell free work has shown that aSyn and Tau induce each other’s fibrillization^29^, and that aSyn is able to interact with Tau by binding of its C-terminal region to the microtubule binding domain (MTBD) of Tau^27,30^. Thus, there is a growing body of evidence supporting that aSyn and Tau are able to interact and influence each other. However, how the interaction between aSyn and Tau enhances neurodegeneration is still poorly understood.

Bimolecular Fluorescence Complementation (BiFC) is a technique widely used to examine protein–protein interactions. It is based on the structural complementation of two halves of a fluorescent protein, allowing direct visualization of interacting proteins. In neurodegenerative diseases, this technique has been used to monitor the oligomerization of aSyn^31–35^, Tau^34,36^, Huntingtin^37,38^, DJ-1^39,40^, and TDP-43^41^ in vitro, as well as to study self-interaction of aSyn^42,43^ and Tau^44^ in vivo.

The aim of the present study was to assess the ability of aSyn and Tau to interact with each other, and to evaluate whether BiFC is a suitable technique to monitor aSyn-Tau interaction in vitro and in vivo. We concluded that aSyn and Tau are able to interact with each other and that the BiFC assay is an effective tool to study aSyn-Tau interaction in vitro and in vivo, providing a valuable tool for examining the pathological and physiological consequences of aSyn-Tau interaction, and facilitating the screening of potential drugs that could boost or inhibit this interaction.

## Results

### aSyn-Tau interaction in cell lines

**Figure 1:**
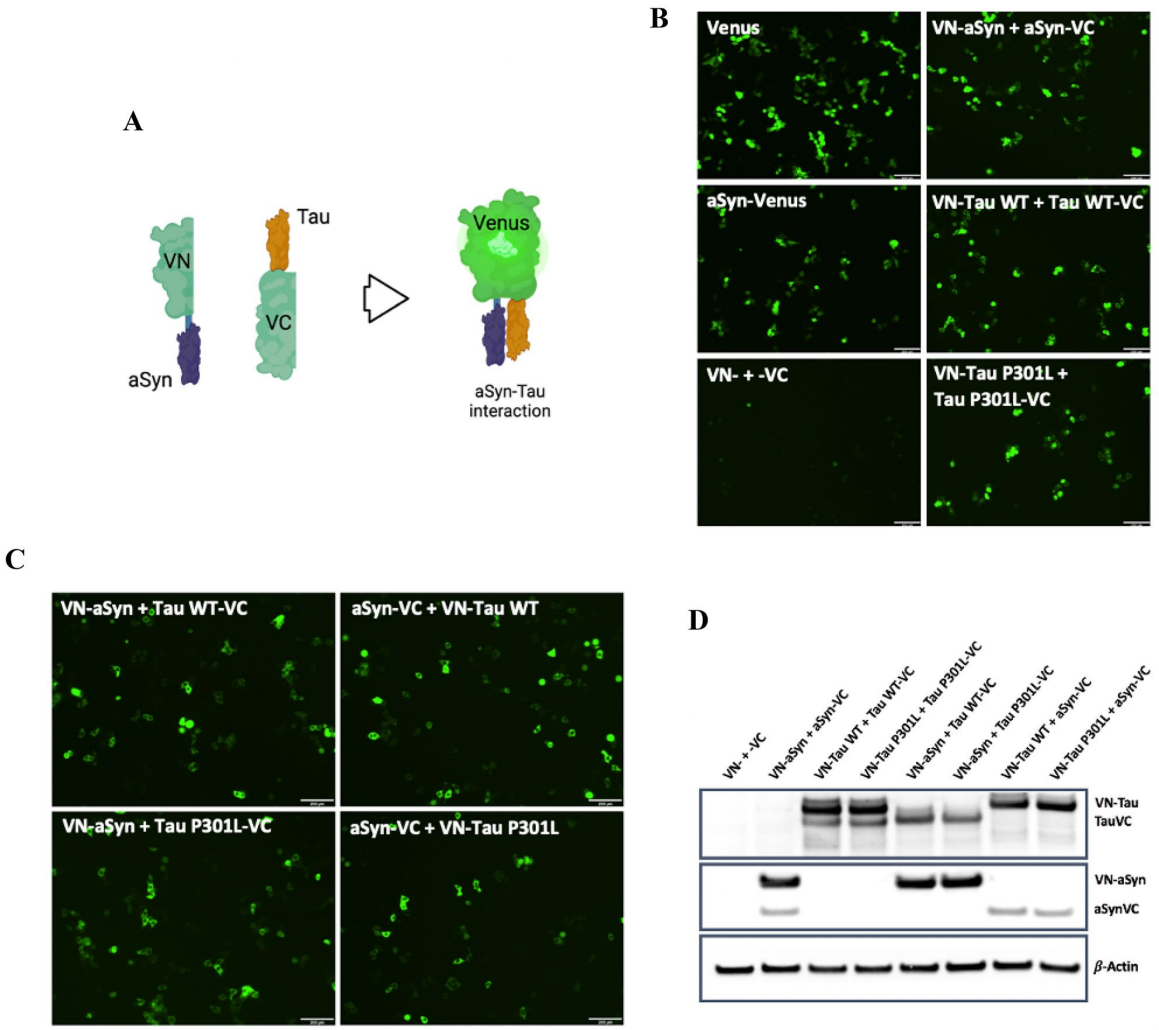
Bimolecular Fluorescence Complementation (BiFC) assay confirms aSyn-Tau interaction in HEK293 cells. (**A**) Schematic representation of the BiFC assay. aSyn WT and/or Tau (WT or P301L) were expressed fused to one of the halves of the protein Venus (VN- or -VC). Complementation of the Venus halves due to interaction of aSyn with Tau promotes changes in the molecular conformation of the Venus protein leading to its emission of fluorescence (scheme created with BioRender.com). (**B**) Fluorescence emitted by Venus complementation 24 h post-transfection in HEK293 cells shows self-interaction of aSyn, Tau WT and Tau P301L. HEK293 cells were transiently (co-)transfected with different combinations of the BiFC constructs; the fluorescence emitted by Venus expression or complementation was recorded 24 h after transfection. Scale bar: 200 µm. (**C)** Fluorescence emitted by Venus complementation 24 h post-transfection in HEK293 cells shows interaction of aSyn with Tau (WT and P301L). HEK293 cells were transiently co-transfected with different combinations of the aSyn and Tau constructs, and the fluorescence emitted by Venus complementation was recorded 24 h after transfection. Scale bar: 200 µm. (**D**) Total levels of Tau and aSyn measured by western blot (WB). The levels of expression of aSyn and Tau in the cell lysate of HEK293 cells co-transfected with different BiFC constructs were measured by WB against total Tau and aSyn. β-Actin was used as loading control.

The BiFC assay was chosen as a tool to evaluate aSyn-Tau interaction. aSyn (WT) and Tau (WT and P301L) were expressed fused to one of the halves of the Venus protein. After interaction of the two proteins of interest, the presence of both Venus halves in close proximity induces a conformational change in their structure that leads to their complementation and the emission of fluorescence that can be detected under a fluorescence microscope (Fig. 1A).

To examine whether aSyn-Tau interaction can be detected by the BiFC assay, we (co-) transfected HEK293 cells with different combinations of the BiFC constructs (Table S1). Single transfection of full-length Venus allowed us to determine the maximum level of Venus signal, whereas single transfection of aSyn-Venus confirmed that full-length Venus is able to emit fluorescence when linked to aSyn (Fig. 1B). On the other hand, co-transfection of the Venus halves (VN- and -VC) without aSyn or Tau linked proved that the Venus halves were not able to spontaneously complement with each other in the absence of aSyn and Tau (Fig. 1B). Similar results were obtained when VN-aSyn or VN-Tau WT were co-expressed with the empty -VC half. However, the expression of the empty VN- half together with aSyn-VC or Tau WT-VC gave rise to weak fluorescent signal (Fig. S1A).

Next, we used the BiFC assay to assess the self-interactions of aSyn, Tau WT and Tau P301L. HEK293 cells were co-transfected with paired BiFC constructs: VN-aSyn + aSyn-VC, VN-Tau WT + Tau WT-VC and VN-Tau P301L + Tau P301L-VC. These co-transfections resulted in the emergence of fluorescence-positive cells (Fig. 1B), reflecting the interaction of the proteins. Thus, we confirmed that self-interactions of aSyn, Tau WT and Tau P301L can each be observed by BiFC in HEK293 cells.

To evaluate aSyn-Tau (WT and P301L) interactions, HEK293 cells were co-transfected with aSyn and Tau (WT or P301L) linked to complementary Venus halves: VN-aSyn + Tau WT-VC, VN-Tau WT + aSyn-VC, VN-aSyn + Tau P301L-VC and VN-Tau P301L + aSyn-VC. In all the combinations, we observed the complementation of the Venus protein, reflecting the interaction of aSyn and Tau (Fig. 1C). The fluorescence obtained by complementation of the Venus protein was similar to the levels observed when evaluating the self-interactions of aSyn, Tau WT and Tau P301L. This demonstrates that aSyn-Tau (WT and P301L) interaction can be monitored in vitro by BiFC.

Prior work has shown differences in the levels of expression of the different BiFC constructs. To determine the protein levels upon transfection with the BiFC constructs in our study, HEK293 cells were co-transfected with different combinations of the BiFC constructs and 24 h post-transfection aSyn and Tau levels were evaluated. We observed that the protein levels of aSyn and Tau were higher when they were linked to the VN- half than when linked to the -VC half (Fig. 1D, S1B and C).

**Figure 2:**
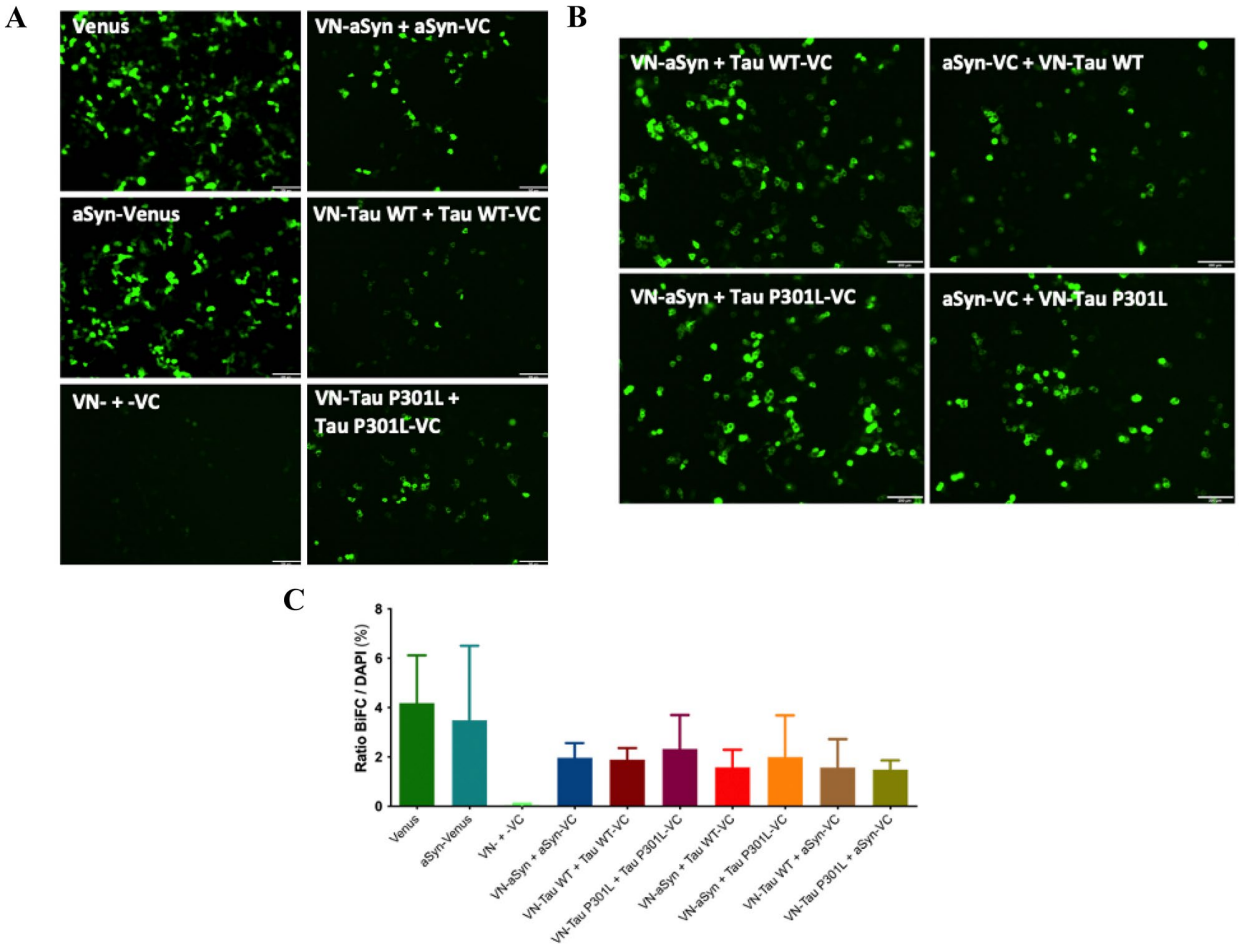
aSyn-Tau interaction in SH-SY5Y human neuroblastoma cells. (**A**) Fluorescence emitted by Venus complementation 24 h post-transfection in SH-SY5Y cells shows self-interaction of aSyn, Tau WT and Tau P301L. SH-SY5Y cells were transiently (co-)transfected with different combinations of the BiFC constructs, and the fluorescence emitted by Venus expression or complementation was recorded 24 h after transfection. Scale bar: 200 µm. (**B**) Fluorescence emitted by Venus complementation 24 h post-transfection in SH-SY5Y cells shows interaction of aSyn with Tau (WT and P301L). SH-SY5Y cells were transiently co-transfected with different combinations of the aSyn and Tau constructs, and the fluorescence emitted by Venus complementation was recorded 24 h after transfection. Scale bar: 200 µm. (**C**) BiFC interaction in (co-)transfected SH-SY5Y cells after 24 h. The percentage of fluorescent (BiFC positive) SH-SY5Y cells in relation to the total number of cells present in the FOV was quantified. Data are expressed as mean ± SD. n = 3.

To test whether aSyn-Tau interaction can also be observed in a neuron-like cell, we (co-) transfected SH-SY5Y (human neuroblastoma) cells with the different combinations of the BiFC constructs (Table S1). As observed in HEK293 cells, the single transfection of Venus led to full Venus fluorescence, while aSyn-Venus expression demonstrated the ability of Venus to emit fluorescence when linked to aSyn (Fig. 2A). Additionally, the co-transfection of VN-, VN-aSyn or VN-Tau WT together with the empty -VC half showed no fluorescence, whilst we observed considerable fluorescence when co-expressing the empty VN- half together with aSyn-VC or Tau WT-VC (Fig. S2A). In SH-SY5Y cells, aSyn, Tau WT and Tau P301L were also able to self-interact (Fig. 2A), and we were able to detect complementation of Venus when aSyn and Tau (WT or P301L) were co-transfected (Fig. 2B). This shows that aSyn-Tau (WT and P301L) interaction can be observed in vitro by BiFC in a human neuroblastoma cell line.

To explore the dynamic processes of the interaction, Venus complementation in SH-SY5Y cells was examined by time-lapse, live-cell imaging every 30 min after transfection for 24 h. For each condition, a field of view (FOV) was chosen and tracked, and changes in fluorescence intensity over time in the cells within the FOV were measured. We observed initial fluorescence signal at different time points between conditions. Full-length Venus was the first in emitting fluorescence (~ 7.5 h), while fluorescence of aSyn-Venus took a longer time to appear (~ 11 h); however, both conditions showed similar intensity over time. Self-Interactions of aSyn, Tau WT, and Tau P301L showed similar initiation times (9–11 h) and progression, with the aSyn coupling exhibiting the strongest complemented fluorescence (Fig. S2B, upper graph). When aSyn and Tau (WT or P301L) were co-transfected, we could observe that the aSyn-Tau P301L couplings showed earlier initiation time but lower complemented fluorescence intensity than the aSyn-Tau WT couplings (Fig. S2B, lower graph).

Considering that the degree of interaction between the proteins in one cell could be influenced by the amount of plasmid(s) transfected into the cell and the levels of transgene expression, which could affect the dynamics of the interaction over time, we took a second approach to have a better picture of potential differences in the interaction between constructs. SH-SY5Y cells were (co-)transfected and after 24 h the percentage of fluorescent cells in relation to the total number of cells present in the FOV was quantified. This quantification showed no significant differences between aSyn, Tau WT, and Tau P301L when they were expressed by themselves or in combination. The percentage of fluorescent cells obtained after co-transfection of the different couplings was not significantly different to that obtained after single transfection of Venus or aSyn-Venus (Fig. 2C), while the fluorescence emitted by VN- + -VC coupling was undetectable.

### Direct interaction of aSyn-Tau in mouse SNpc

Next, we asked whether the physiological environment of the living mouse brain would enable the detection of the aSyn-Tau interaction using BiFC. For that, AAV6 carrying different BiFC constructs (Table S2) were injected into the SNpc of 10–12-week-old WT mice, and 15 weeks post-injection complemented Venus fluorescence was evaluated. Low AAV titer was used to detect potential aSyn-Tau interactions without reaching toxicity. The 15 weeks time point was chosen due to that after AAV injection, the full expression of the transduced proteins in the brain takes up to 3–4 weeks. Moreover, previous publications showed self-interaction of Tau^44^ or aSyn^42^ by BiFC complementation after 12 weeks, thus, supporting that 15–16 weeks would be a good time frame for studying aSyn-Tau interaction.

**Figure 3:**
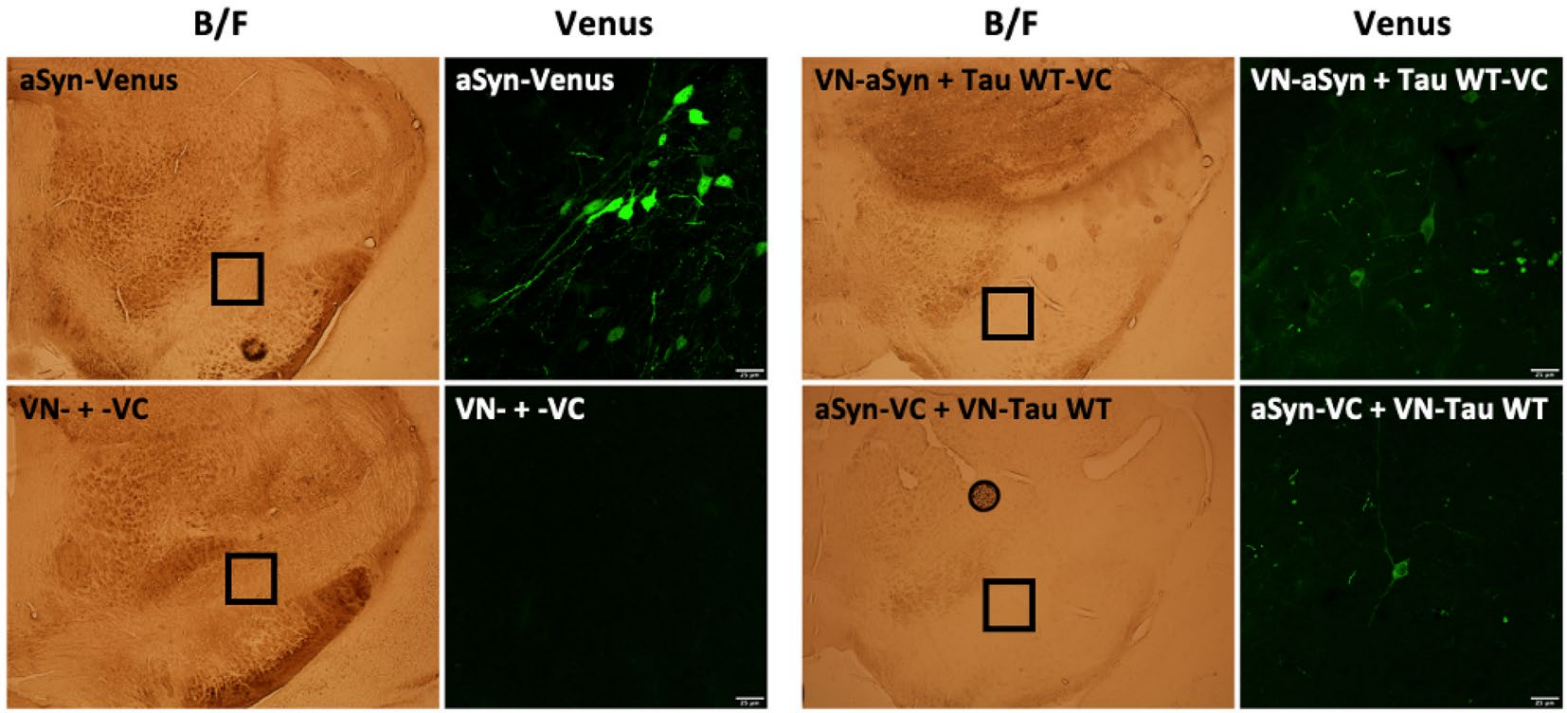
aSyn-Tau interaction in mouse substantia nigra pars compacta (SNpc). BiFC interaction in mouse SNpc. 10–12-week-old mice were injected in SNpc with AAV6 carrying different BiFC constructs. After 15 weeks, Venus fluorescence was evaluated. The expression of aSyn fused to full-length Venus provided the maximum level of fluorescence expected. The interaction of aSyn and Tau was reflected by Venus complementation in VN-aSyn + Tau WT-VC and VN-Tau WT + aSyn-VC injected animals. As expected, the expression of the Venus halves not linked to proteins did not produce any fluorescence in the injected area. Boxed area on Bright Field (B/F) images is magnified to the right. Scale bar: 25 µm.

The expression of aSyn fused to full-length Venus provided the highest level of fluorescence. This expression was confined to a few neurons. As expected, and in agreement with the in vitro results, the expression of the Venus halves (VN- + -VC) not linked to interacting proteins did not produce any fluorescence in the injected area (Fig. 3). Similar results were observed when VN-aSyn or VN-Tau WT were co-expressed with the empty -VC half. However, the expression of the empty VN- half together with aSyn-VC or Tau WT-VC gave rise to a background residual fluorescence (Fig. S3A). Remarkably, the co-expression of aSyn and Tau (VN-aSyn + Tau WT-VC and VN-Tau WT + aSyn-VC) led to Venus complementation (Fig. 3), reflecting the interaction between aSyn and Tau in the SNpc of mice. Therefore, BiFC is shown to be a suitable technique to monitor aSyn-Tau interaction in vivo.

Further, we evaluated whether aSyn-Tau interaction could lead to toxicity in our system. For that, tyrosine hydroxylase (TH) immunolabelling was performed in mice injected with aSyn-Venus, VN- + -VC, VN-aSyn + Tau WT-VC, and aSyn-VC + VN-Tau WT (Fig. S3B). In general, the expression of the BiFC constructs did not seem to affect the SNpc; however, tissue damage associated with glass capillary tract injury was eventually observed, as can be noticed in aSyn-Venus injection (Fig. S3B). Moreover, rotarod and amphetamine-induced rotation tests were performed in the animals 15 weeks after the injection of the different AAV6. No significant differences were found between groups (data not shown), potentially due to the low number of neurons that expressed the BiFC constructs.

### Direct interaction of aSyn-Tau in the rat SNpc

**Figure 4:**
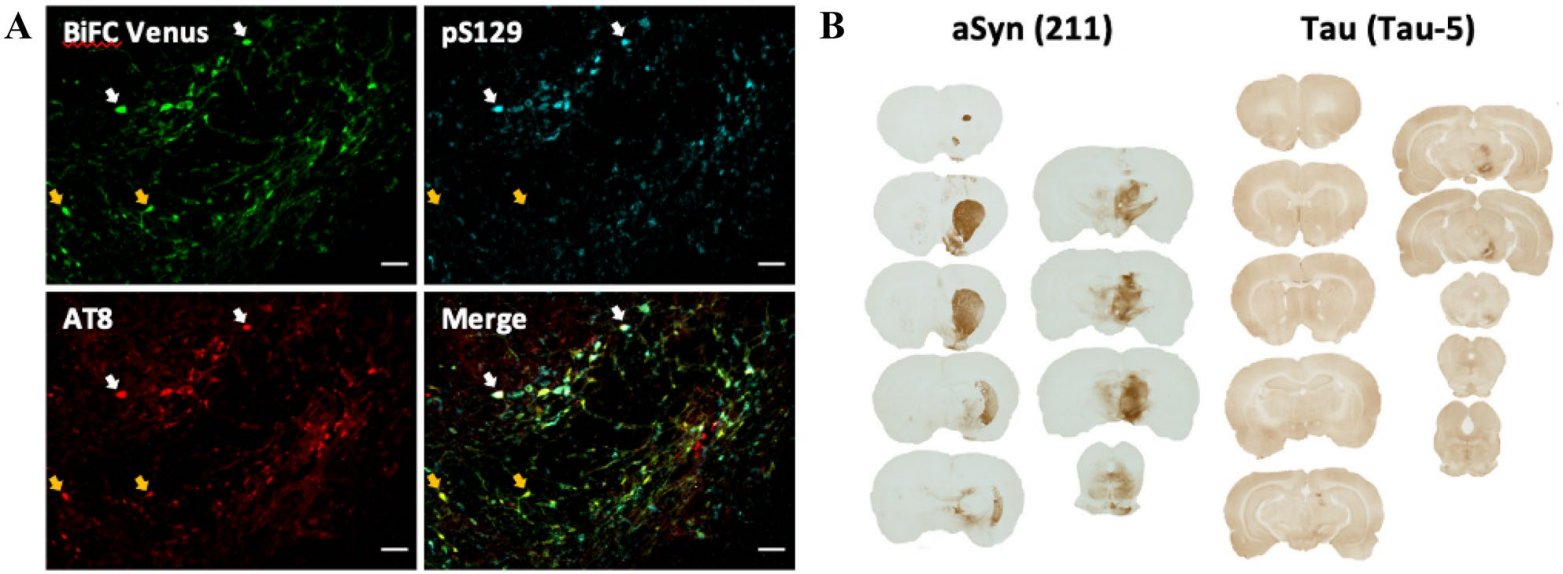
Molecular characterization of aSyn-Tau interaction in rat SNpc. aSyn-Tau interaction in rat SNpc. Eight weeks after co-injection of VN-aSyn and Tau WT-VC in rat SNpc immunohistochemistry was performed. (**A**) Immunohistochemistry for phosphorylated epitopes of aSyn (pS129) and Tau (AT8) suggests that aSyn and Tau can be phosphorylated while they are interacting with each other. Scale bar: 50 µm (**B**) Distribution of aSyn and Tau after injection of VN-aSyn and Tau WT-VC in rat SNpc. aSyn distribution shows a clear transport of aSyn from the injected area to the striatum. In contrast, the expression of human Tau seems to be confined to the SNpc.

To corroborate that the BiFC assay can be used to visualize aSyn-Tau interaction in a different animal model, we injected AAV6 carrying VN-aSyn + Tau WT-VC, the aSyn-Tau combination that yielded the strongest interaction in our mouse system, in the rat SNpc. In this case, due to the lack of toxicity induced by AAV6 and based on the results obtained in mice, we increased the viral load 10 times. After 8 weeks, Venus complementation resulting from aSyn-Tau interaction was observed (Fig. 4A), confirming that the BiFC assay can be used in vivo in different rodent models. This complementation was observed both in SNpc (Fig. 4A) and in the striatum (Fig. S4), supporting that the interaction between aSyn and Tau is not only occurring in the soma of the transduced neurons but also at the terminal fields of their axons.

To characterize the state of aSyn and Tau, immunohistochemistry against phosphorylated forms of aSyn and Tau was performed. AT8 immunostaining showed that Tau was substantially phosphorylated at its S202 and T205 epitopes when co-expressed together with aSyn (Fig. 4A), consistent with prior work that showed that aSyn is able to induce Tau phosphorylation^1,22,23,27^. In addition, aSyn was also phosphorylated at its S129 epitope. Interestingly, we observed differential co-localization between complemented (BiFC) Venus, AT8-positive and pS129-positive profiles. It appeared that most of the neuronal cell bodies positive for BiFC Venus are also positive for both AT8 and pS129 (Fig. 4A, white arrows), suggesting that both Tau and aSyn can be phosphorylated while they are interacting with each other. A few neuronal profiles with BiFC Venus exhibited either AT8- or pS129-positivity (Fig. 4A, orange arrows), implying that interacted Tau and aSyn may be phosphorylated to different extents. Furthermore, and unlike for Venus complementation, we did not observe phosphorylation of aSyn or Tau in the striatum.

Lastly, immunohistochemistry against total aSyn and total Tau was carried out to determine the distribution of the injected aSyn and Tau in the brain. Interestingly, marked propagation of the human aSyn was observed, indicative of transport of the protein from SNpc to the striatum. In contrast, the expression of human Tau remained mainly confined to the SNpc (Fig. 4B).

## Discussion

aSyn is a phospholipid-binding protein with a role in vesicle trafficking and neurotransmitter release^45^. Tau is a microtubule-associated protein involved in microtubule stabilization and axonal outgrowth^46,47^. Both are soluble and natively unfolded proteins^48,49^, that present common mechanisms of transmission^50^, and are characterized by their pathological aggregation in neurodegenerative disorders. aSyn and Tau accumulation leads to the formation of oligomeric and fibrillar forms. A growing body of evidence points to an intertwined interaction between aSyn and Tau in pathology. Whilst patient-derived brain tissue exhibits co-occurrence of aSyn and Tau pathology in different neurodegenerative diseases, studies carried out in animal models show that aSyn and Tau interaction increases neurotoxicity. However, how aSyn and Tau influence each other, and how their interaction enhances neurodegeneration is still poorly understood.

One of the limitations of studying the consequences of aSyn-Tau interaction is the limited number of techniques available to monitor protein interaction in vivo. We applied the BiFC assay as a potential tool to visualize aSyn-Tau interaction. BiFC has been used by several groups aiming to obtain a direct visualization of protein oligomerization due to the relevance of oligomer formation in neurodegenerative disorders. In synucleinopathies and tauopathies, the presence and propagation of oligomeric forms of aSyn and Tau, respectively, correlates better than fibril formation with disease pathogenesis^45,51^.

Previously, the direct interaction between aSyn and Tau has been demonstrated in cell free assays^27,30^. Thus, in the present study we aimed to assess the ability of aSyn and Tau to interact with each other in a biologically relevant context, and to evaluate whether BiFC is a suitable technique to monitor aSyn-Tau interaction in vitro and in vivo.

We showed that BiFC is able to track aSyn-Tau (WT and P301L) interaction in vitro in HEK293 cells and in a neuron-like cell line, SH-SY5Y cells. After confirming the functionality of this assay to detect aSyn-Tau interaction in vitro, in vivo injections in SNpc of WT mice and rats were performed. In mice, injections with low titers of AAV6 vectors carrying the different BiFC constructs revealed that aSyn-Tau interaction could be observed in vivo by BiFC, without inducing toxicity. Interestingly, we observed a difference in the interaction between aSyn-Tau in relation to where the Venus halves were placed, resulting in greater interaction when VN-aSyn + Tau WT-VC were co-expressed than when aSyn-VC + Tau WT-VN were co-expressed. Next to the interaction between aSyn and Tau, we observed unexpected fluorescence when empty VN- was co-expressed with aSyn-VC or Tau WT-VC, which did not happen when the proteins linked to the N-terminal Venus half (VN-aSyn or VN-Tau WT) were co-expressed with the empty -VC construct. This phenomenon has been previously reported in the literature^33,37,52^.

Previous studies have explored the potential of BiFC to visualize protein interactions, however, some limitations of this assay need to be kept in mind. An imbalance in the levels of expression of the BiFC constructs has been long reported in the literature^31–38,40,43,52,53^, suggesting an increase in the stability or decrease in the degradation of the VN- halves in comparison with -VC halves^35^. Interestingly, Frey et al. (2020) addressed this imbalance in protein levels. They developed a bidirectional expression system that allowed for the inducible expression of both BiFC constructs (VN-aSyn and aSyn-VC) under the control of a single promoter. Nevertheless, even with this system, the imbalance in protein levels was seen, with VN-aSyn expression being higher than aSyn-VC expression. In our study we observed a similar phenomenon, that could potentially explain the propensity of the empty VN- half to interact with aSyn-VC and Tau-VC. Theoretically, the empty BiFC constructs (VN- and -VC) should not be able to complement when the interacting proteins are not present, yet, the presence of background fluorescence due to the interaction of the empty VN- construct is described^33,37,52^.

The validity of the BiFC assay to monitor protein interaction has been tested by several groups. Robida et al. (2009) showed the ability of the BiFC assay to visualize protein interaction when this interaction had to be induced by the presence of a ligand^52^. In addition, point mutations in the amino acids involved in the interaction between proteins led to an inhibition of Venus complementation^39,53–55^. Moreover, a recent study showed that even when the difference in the levels of expression of the BiFC constructs could lead to an increase in the self-aggregation of the overexpressed ones, the proteins of interest were able to physically interact with each other and this could be detected by BiFC^35^. Thus, the BiFC assay is suitable to determine protein–protein interactions but it is not sensitive enough to distinguish the degree of oligomerization between the interacting proteins^35^. This supports that the BiFC assay can be used to visualize aSyn-Tau interaction.

When VN-aSyn and Tau WT-VC AAVs were injected together in the SNpc of rats, we could observe that the co-expression of the proteins led to high levels of Tau phosphorylation at its S202 and T205 epitopes. Previous studies have shown the ability of aSyn to induce Tau phosphorylation^1,22,23,27^. Similarly, aSyn was phosphorylated at its pS129 epitope when co-expressed with Tau, although to a lesser extent.

In AD and PD, protein aggregation and neurodegeneration appear to follow a spatiotemporal progression^56,57^, that suggests cell-to-cell transfer of pathology^58–60^. This is supported by the spreading of aSyn aggregates from the host to healthy transplanted neurons observed in postmortem tissue of PD patients^61,62^. In our study, after co-injection of VN-aSyn and Tau WT-VC in rat SNpc, we detected evidence consistent with transport of human aSyn from SNpc to the striatum, in contrast, there was much less transport of human Tau. However, the transport of Tau to the terminal fields of the axons was supported by the presence of Venus complementation in the striatum. Nevertheless, the difference in the propagation of aSyn and Tau in this study may be caused by, amongst others, the different size of aSyn and Tau molecules or by the different levels of expression of the proteins.

We conclude that aSyn and Tau are able to interact in a biological setting, and that the BiFC assay is an effective tool to study aSyn-Tau interaction in vitro in cells and in different rodent models in vivo, providing a new resource for examining the pathological and physiological consequences of aSyn-Tau interaction. More work needs to be done to understand the impact of this interaction, and the consequences of its inhibition and/or facilitation in neurodegeneration. For example, future work in primary neuronal cultures could be of great value to better understand the mechanism(s) by which aSyn and Tau are able to interact with each other and the implications of this interaction in neuronal degeneration.

## Materials and methods

### Animal work: mice and rats

C57BL/6J mice and Sprague Dawley rats were purchased from Taconic (Ejby, Denmark). Animals were randomly assigned to each group. All experiments were approved and performed following the guidelines approved by the Malmö/Lund Ethics Committee, ethical permits number: M72-2016 and M73-2016. The reporting of the animal work performed in the manuscript follows the recommendations in the ARRIVE guidelines.

### Generation of constructs and AAV production

The BiFC Tau constructs, VN-Tau (WT and P301L) and Tau-VC (WT and P301L), were generated by enzymatic restriction and ligation. Venus N-terminal (VN-) and Venus C-terminal (-VC) backbones were obtained from the VN-aSyn and aSyn-VC constructs previously described by Outeiro *et al.* (2008). Tau WT and Tau P301L sequences (0N4R isoform) were amplified from pRK5-EGFP-Tau (Addgene plasmid #46904) and pRK5-EGFP-Tau P301L (Addgene plasmid #46908)^63^, plasmids gift of Dr. Karen Ashe. In brief, Tau sequences were amplified by PCR (DreamTaq PCR Master Mix, Thermo Fisher Scientific). The Venus halves (VN- and -VC) were used as a backbone. The backbones and the amplified Tau sequences were cleaved by AfIII (#ER0831, Thermo Fisher Scientific) and XhoI (#ER0691, Thermo Fisher Scientific) restriction enzymes, and ligated by incubation with T4 DNA ligase (#EL0014, Thermo Fisher Scientific). The generated constructs were confirmed by sequencing. From these constructs, Adeno-Associated Virus Type 6 (AAV6) were produced in the AAV Vector Lab at the MultiPark core facility at Lund University.

### Stereotaxic AAV Injections

Animals were unilaterally injected with a final volume of 2 µl containing different combinations of the BiFC AAVs. Vector solutions were injected using a 5 µl Hamilton syringe fitted with a glass capillary. The injection was performed manually in the case of mice, or by means of a pump with an infusion rate of 0.2 µl/min in rats. After delivery, the needle was left in place for 5 min before retraction.

For mice, 10–12-week-old mice were injected in SNpc (stereotaxic coordinates from bregma: AP −2.7 mm, ML +1.5 mm, DV −4.2 mm) with AAV6 carrying the BiFC constructs (listed in Table S2) expressed under the control of the Synapsin1 (Syn1) promoter, at a final injection titer of 1x10^12^ genome copies/ml (gc/ml). After 15 weeks, animals were sacrificed.

For rats, 8–10-week-old rats were injected in SNpc (stereotaxic coordinates from bregma: AP −5.3 mm, ML −1.7 mm, DV −7.2 mm) with AAV6 carrying VN-aSyn and Tau WT-VC constructs expressed under the control of the Syn1 promoter; 5 × 10^13^ gc/ml were injected. Eight weeks after injection, animals were sacrificed.

### Cell culture and transfection

#### Human embryonic kidney 293 (HEK293) cell line

HEK293 cells (#85120602, Sigma-Aldrich) were cultured in Gibco DMEM supplemented with 10% Fetal Bovine Serum (FBS) and 1% Penicillin-Streptomycin (P/S), all from Thermo Fisher Scientific, in a humidified atmosphere at 5% CO_2_ at 37 °C. For immunocytochemistry, cells were plated in a 48-well plate, and 0.1 µg of the corresponding DNA was delivered per well along with 1 µl of Lipofectamine 2000 (#11668019, Thermo Fisher Scientific), following the manufacturer’s protocol. HEK293 cells were transfected at 60-70% confluency, and 24 h post-transfection they were fixed with 4% paraformaldehyde (PFA) for 15 min. For western blot, cells were plated in a 12-well plate, and 0.4 µg of DNA was delivered per well along with 4 µl of Lipofectamine 2000. After 24 h cells were lysed.

#### Human neuroblastoma (SH-SY5Y) cell line

SH-SY5Y cells (#94030304, Sigma-Aldrich) were grown in Gibco DMEM supplemented with 10% FBS and 1% P/S. Cells were incubated in a humidified atmosphere at 5% CO_2_ at 37 °C. For differentiation, plates were coated with collagen G (1:20; L7213, Biochrom) for 2 h, and cells plated at a concentration of 10.526 cells per cm^2^. The following 4 days cells were incubated in FBS free medium supplemented with 10 µM retinoic acid (R2625, Sigma-Aldrich). After that, cells were incubated in FBS free medium supplemented with 50 ng/ml BDNF (212-GD, R&D Systems). For immunocytochemistry, cells were plated in a 48-well plate, and 0.1 µg of the corresponding DNA was delivered per well along with 1 µl of Lipofectamine 2000, following the manufacturer’s protocol. SH-SY5Y cells were transfected, and live-cell imaged every 30 min for 24 h when evaluating the time-dependent interaction of the BiFC constructs. For quantification of the ratio of BiFC positive cells, SH-SY5Y were fixed 24 h post-transfection.

### Western blot

HEK293 were co-transfected with different combinations of BiFC constructs (Table S1). After 24 h, cells were lysed in Pierce RIPA buffer (#89900, Thermo Fisher Scientific) containing protease inhibitor (1:100; #78430, Thermo Fisher Scientific) and phosphatase inhibitor (1:100; #11833955, Thermo Fisher Scientific). Protein concentration was measured by BCA assay (#23227, Thermo Fisher Scientific), and 5 µg of protein per sample were denatured at 95 °C for 5 min. Proteins were separated in NuPAGE 4-12% Bis-Tris gels (#NP0321PK2, Thermo Fisher Scientific), and then transferred to a nitrocellulose membrane (#IB23001, Invitrogen) using an iBlot 2 gel transfer device (Invitrogen). The membrane was blocked in 5% Bovine Serum Albumin (BSA; #A7906, Sigma-Aldrich) for 1 h, and cut at 38 kDa prior to the incubation with antibodies. The lower part of the membrane was incubated overnight in primary antibody against aSyn, while the upper membrane was incubated in total Tau or β-Actin (Table S3) overnight. After that, membranes were washed in PBS with 0.05% Tween (PBS-T), incubated in anti-mouse or anti-rabbit horseradish peroxidase (HRP) secondary antibody for 1 h, washed in PBS-T, incubated in enhanced chemiluminescence (ECL) substrate (#1705061, BioRad) and developed in a ChemiDoc MP imager (BioRad).

### Live-cell Imaging and Quantification

After 5 days of differentiation, SH-SY5Y cells were (co-)transfected with different combinations of BiFC constructs (Table S1). After transfection, Venus complementation in SH-SY5Y cells was recorded by live-cell imaging every 30 min for 24 h. Images were taken in a Nikon Eclipse Ti microscope under a 10x objective. For each condition, a field of view (FOV) was chosen and tracked, changes in fluorescence intensity in the cells within the FOV with time were measured. The corrected fluorescence was calculated and divided within the total amount of BiFC positive cells present in the FOV after the 24 h:

$$\frac{\mathrm{Total \,\,fluorescence}-\mathrm{Initial\,\, background\,\, fluorescence}}{\mathrm{Total\,\, BiFC\,\, positive\,\, cells}}$$

Alternatively, 24 h after transfection, SH-SY5Y cells were fixed with 4% PFA for 15 min, washed with PBS-T, incubated in DAPI (1:1000; D9542, Sigma-Aldrich) diluted in PBS for 30 min and washed again in PBS-T. Image acquisition was performed using an inverted epifluorescence microscope Olympus BX53, under a 20x objective. After that, the ratio of BiFC positive cells per total amount of cells in the FOV was quantified using Metamorph Premier (Cairn Research Ltd, Kent, UK).

### Immunohistochemistry

Animals were deeply anaesthetized with isoflurane, perfused first with PB-buffer, followed by perfusion with 4% PFA, and the brains extracted. Brains were post-fixed overnight and stored in 0.1 M PBS with 30% sucrose until sectioned. For mouse brain samples, 40 µm sections were washed in PBS, blocked in 5% Normal Donkey Serum (NDS) for 1 h, and incubated in TH primary antibody overnight at room temperature. After incubation, sections were washed in PBS-T and incubated in anti-rabbit secondary antibody for 2 h, washed in PBS-T, and mounted in polyvinyl alcohol mounting medium with DABCO (PVA-DABCO) containing DAPI (1:1000). Venus expression was acquired by using an inverted epifluorescence microscope Nikon Eclipse 80i under a 4x objective, and a Leica SP8 confocal microscope under a 20x and a 40x objective.

For rat brain samples, 40 µm sections were washed in PBS-T, quenched in methanol and H_2_O_2_ (9:1), washed in PBS-T, blocked in 5% BSA for 1 h, and incubated in aSyn (clone 211) or Tau-5 primary antibodies overnight. After incubation, sections were washed in PBS-T and incubated in anti-mouse HRP secondary antibody for 2 h, washed in PBS-T, incubated in ABC-solution (#PK-6100, Vector Laboratories), and washed in PBS-T and incubated in DAB solution (#SK-4100, Vector Laboratories). After that, sections were dehydrated and mounted in dibutyl phthalate in xylene (DPX; #06522, Sigma-Aldrich). For immunofluorescence, the sections were rinsed in TBS, pre-incubated in 10% NDS for 1 h and incubated overnight in pS129 and AT8 primary antibodies. Next, sections were rinsed with TBS, incubated in anti-rabbit and anti-mouse secondary antibodies for 1 h, rinsed with TBS and mounted in PVA-DABCO. After DAB staining, sections were scanned using an EPSON perfection V750 PRO with Silver Fast software. Fluorescence images were acquired in an Olympus BX53 microscope under a 10x objective. See list of antibodies in Table S3.

### Statistical analyses

Data analysis was performed using GraphPad Prism v.9.2. Data were expressed as mean ± SD unless stated otherwise. Kruskal-Wallis test was performed to compare the ratio of BiFC positive cells per total amount of cells in FOV obtained 24 h after (co-) transfection of SH-SY5Y cells. Data was considered statistically significant when *p* value < 0.05 (*p < 0.05, **p < 0.01).

## Supplementary Information

Supplementary Information.

## Data Availability

The datasets generated during the current study are available from the corresponding authors on reasonable request.
