# Supplementary material for: Monitoring the interactions between alpha-synuclein and Tau in vitro and in vivo using bimolecular fluorescence complementation
Source: Sci Rep. 2022 Feb 22;12:2987. doi: 10.1038/s41598-022-06846-9 (PMC8863885; doi:10.1038/s41598-022-06846-9)
Supplement: Supplementary file 1 — Supplementary Information. [file 41598_2022_6846_MOESM1_ESM.pdf]

SUPPLEMENTARY MATERIAL

Supplementary figures

Figure S1. A) Fluorescence emitted by Venus complementation 24 h post-transfection in HEK293 cells. The single transfection with VN-aSyn or aSyn-VC did not produce any fluorescence. Co-expression of VN-aSyn or VN-Tau WT with the empty -VC half led to background fluorescence, while the expression of the empty VN- half together with aSyn-VC or Tau WT-VC gave rise to some fluorescence-positive cells. Scale bar: 200 µm. **B)** Total levels of Tau and aSyn measured by Western blot. The original membrane was cut at 38 kDa (cutting site marked with an arrow) to assess separately the protein levels of aSyn and Tau in the cell lysate of HEK293 cells co-transfected with different BiFC constructs. Stripping of the upper part of the membrane (38-98 kDa) was performed to evaluate the levels of β-Actin (loading control). **C)** High contrast image of the membrane in B) showing total levels of Tau and aSyn measured by Western blot. The original membrane was cut at 38 kDa (cutting sites marked with arrows) to assess separately the protein levels of aSyn and Tau. Stripping of the upper part of the membrane (38-98 kDa) was performed to evaluate the levels of β-Actin. The high contrast allows seeing the edges of the blot.

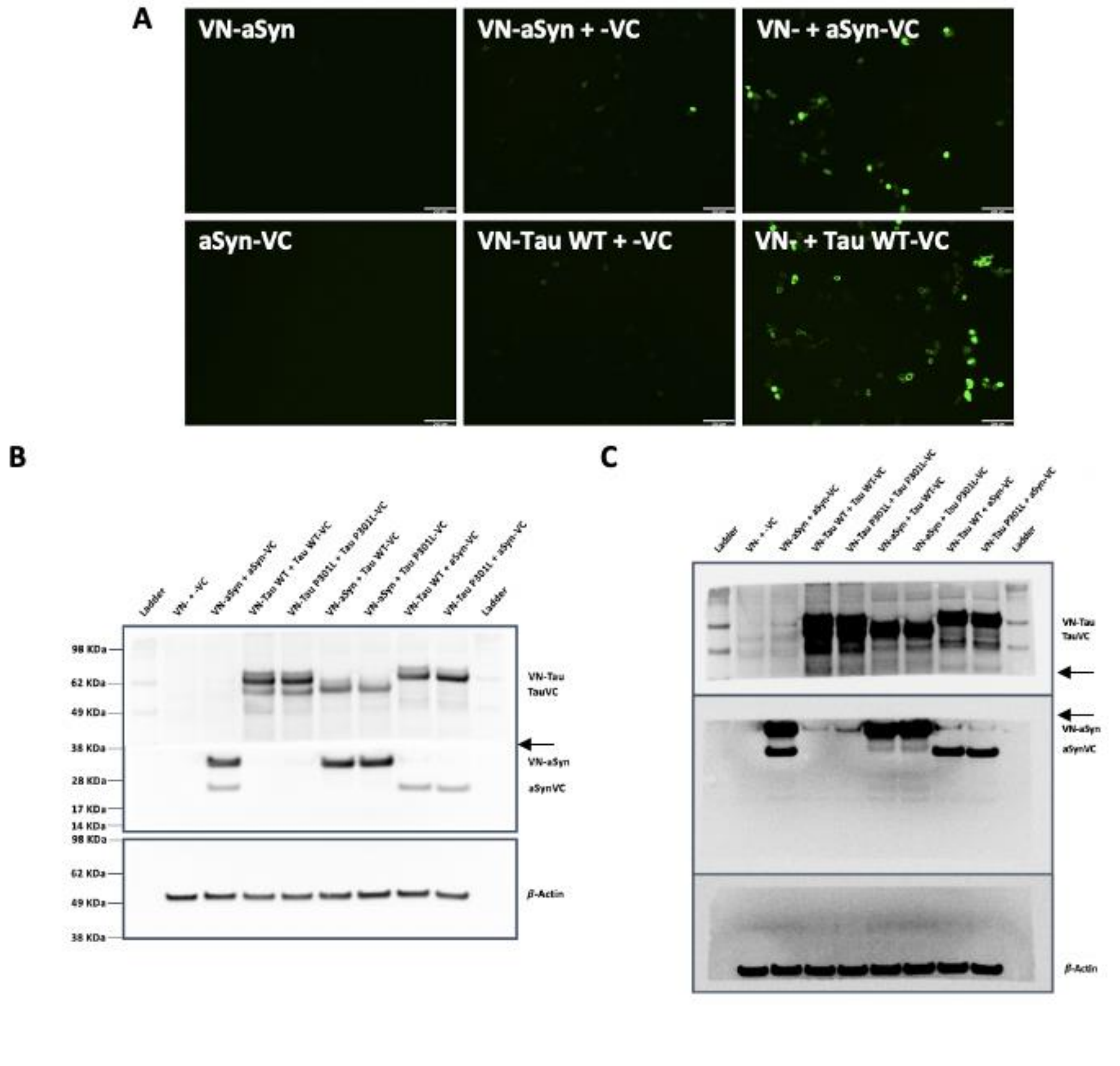

Figure S2. A) Fluorescence emitted by Venus complementation 24 h post-transfection in SH-SY5Y cells. The single transfection with VN-aSyn or aSyn-VC did not produce any fluorescence. Co-expression of VN-aSyn or VN-Tau WT with the empty -VC half led to background fluorescence, while the expression of the empty VN- half together with aSyn-VC or Tau WT-VC gave rise to fluorescence-positive cells. Scale bar: 200 µm. **B)** Time-dependent progressive interaction of BiFC constructs. Protein complementation in SH-SY5Y cells was recorded by live-cell imaging every 30 min after transfection for 24 h. For each condition, a FOV was chosen and tracked, and changes in fluorescence intensity over time in the cells within the FOV were measured. Data obtained every hour is represented. Data are expressed as mean ± SD. n = 2-3.

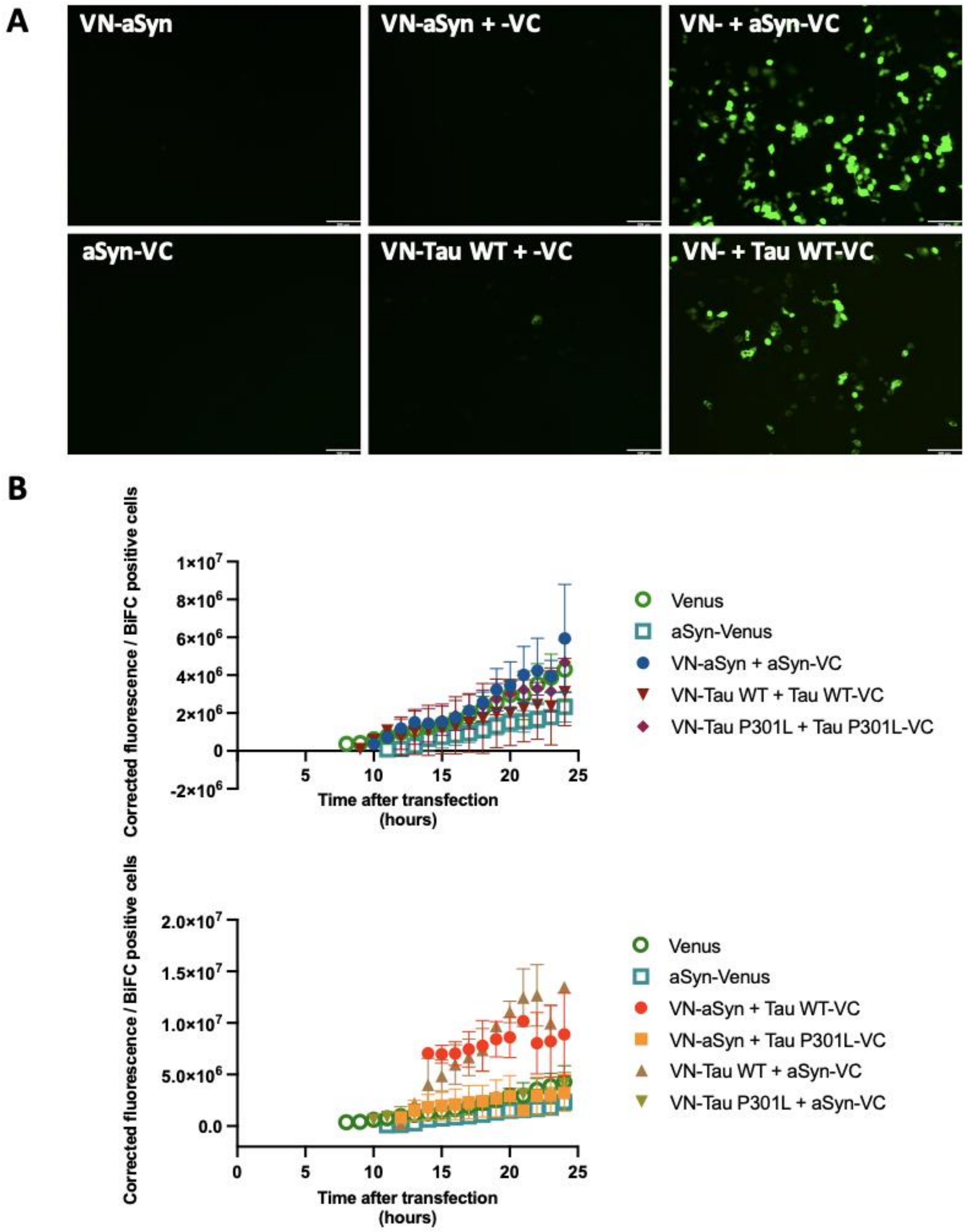

Figure S3. A) BiFC interaction in mouse SNpc. 10-12-weeks-old mice were injected in SNpc with AAV6 carrying different BiFC constructs. After 15 weeks, Venus fluorescence was evaluated. Co-expression of VN-aSyn or VN-Tau WT with the empty -VC half did not produce any fluorescence in the injected area, while the expression of the empty VN- half together with aSyn-VC or Tau WT-VC gave rise to some background fluorescence. Boxed area on Bright Field (B/F) images is magnified to the right. Scale bar: 25 µm. **B)** Tyrosine Hydroxylase (TH) immunolabelling of mice injected with different BiFC constructs in SNpc. BiFC Venus complementation (green) was observed together with TH (magenta) in SNpc. The SNpc did not seem to be affected by the expression of the BiFC constructs; however, mechanical damage to the tissue along the injection tract was eventually observed, as can be noted in the aSyn-Venus injection. Scale bar: 100 µm.

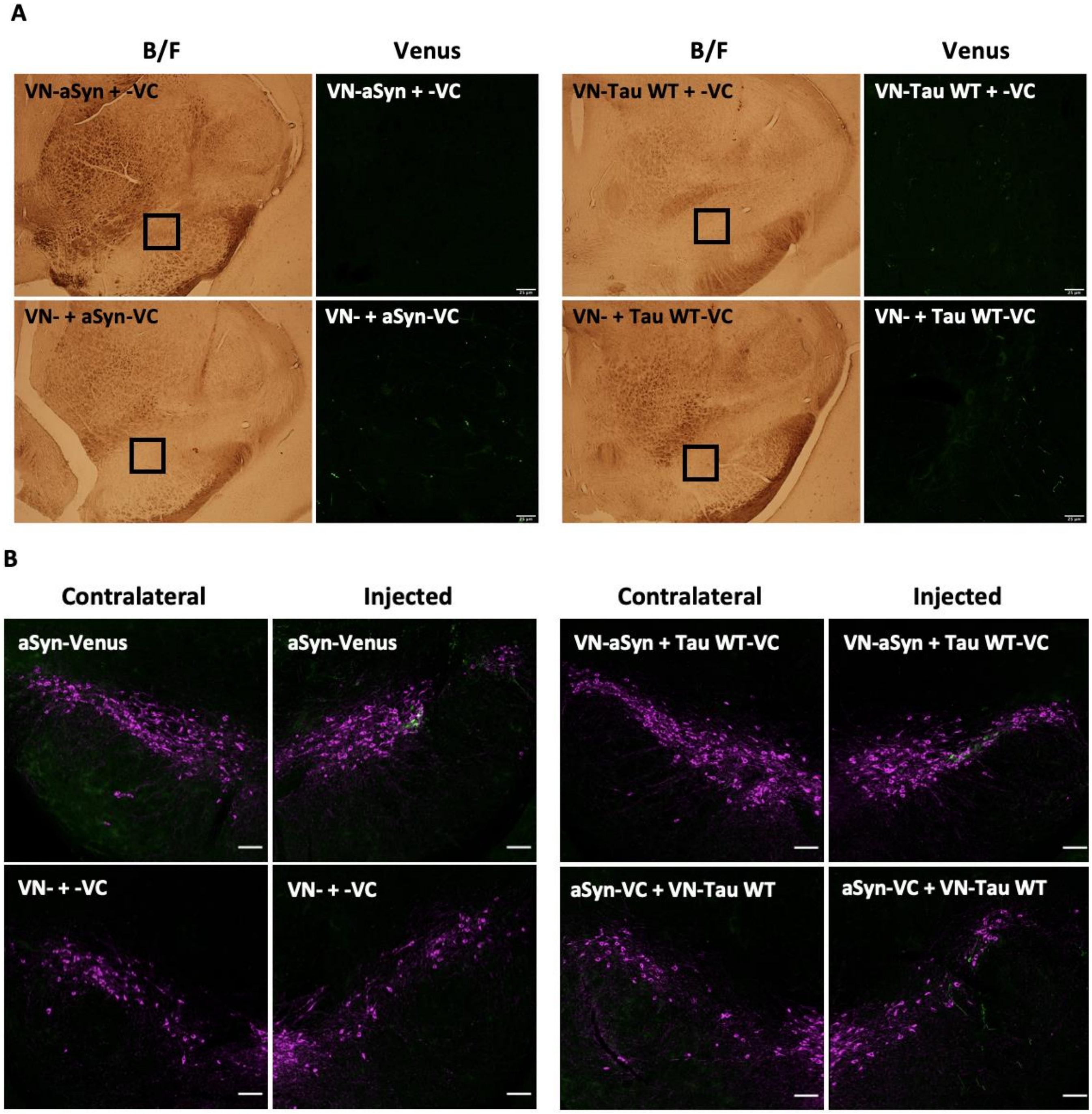

Figure S4. aSyn-Tau interaction in the rat striatum. BiFC Venus complementation was observed in the striatum 8 weeks after the co-injection of VN-aSyn and Tau WT-VC in the rat SNpc. This supports that the interaction between aSyn and Tau is not only happening in the soma of the transduced neurons but also at the terminal fields of their axons. Scale bar: 50 µm

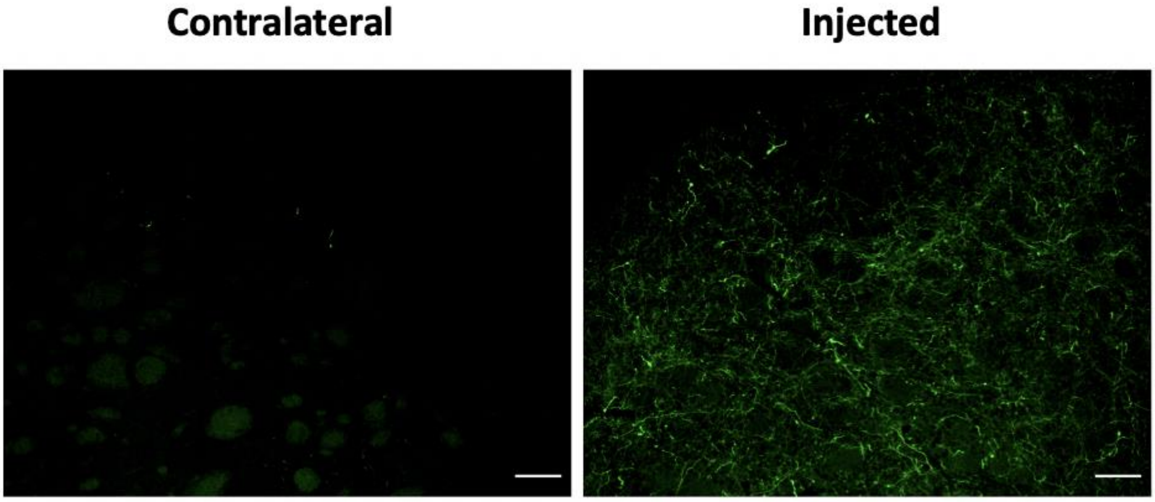

Supplementary tables

Table S1: List of BiFC constructs used in the study.

Construct	Abbreviation
Venus Full-Length (FL)	Venus
α-synuclein – Venus FL	aSyn-Venus
Empty VN-	VN-
Empty -VC	-VC
VN – α-synuclein	VN-aSyn
α-synuclein – VC	aSyn-VC
VN – Tau WT	VN-Tau WT
Tau WT – VC	Tau WT-VC
VN – Tau P301L	VN-Tau P301L
Tau P301L – VC	Tau P301L-VC

Table S2: List of BiFC constructs injected in mice SNpc by AAV6.

Viral vector	Abbreviation
pAAV2-Syn-haSyn-Venus Full	aSyn-Venus
pA2-Syn-empty Venus N	VN-
pA2-Syn-empty Venus C	-VC
pAAV2-Syn-VN-haSyn	VN-aSyn
pAAV2-Syn-haSyn-VC	aSyn-VC
pA2-Syn-VN-TauWT	VN-Tau WT
pA2-Syn-TauWT-VC	Tau WT-VC

Table S3: List of antibodies used in this study

Abbreviation	Antibody	Dilution	Manufacturer	Method
**aSyn**	Purified mouse anti-α-Synuclein	1:1000	610787<br>(BD Biosciences)	WB
**Total Tau**	Anti-Tau antibody	1:1000	ab64193<br>(ABCAM)	WB
**β-Actin**	Monoclonal anti-β-actin antibody	1:4000	A5441<br>(Merck)	WB
**Anti-mouse HRP**	Mouse IgG HRP-conjugated antibody	1:5000	HAF007<br>(R&D Systems)	WB
**Anti-rabbit HRP**	Rabbit IgG HRP-conjugated antibody	1:5000	HAF008<br>(R&D Systems)	WB
**TH**	Rabbit polyclonal antibody against tyrosine hydroxylase	1:1000	AB152<br>(Merck)	IHC
**211**	Mouse monoclonal antibody against total α-Synuclein	1:1000	sc-12767<br>(Santa Cruz Biotechnology)	IHC
**Tau-5**	Mouse monoclonal antibody against total Tau	1:1000	AHB0042<br>(Thermo Fisher Scientific)	IHC
**pS129**	Rabbit monoclonal antibody against α-Synuclein phosphorylated at the S129 epitope	1:1000	AB51253<br>(ABCAM)	IHC
**AT8**	Mouse monoclonal antibody against Tau phosphorylated at S202 and T205 epitopes	1:500	MN1020<br>(Thermo Fisher Scientific)	IHC
**Anti-mouse HRP**	Goat anti-mouse Ig/HRP antibody	1:400	P044701<br>(Dako)	IHC
**Anti-rabbit**	Alexa fluor 647-donkey anti-rabbit IgG	1:400 (pS129)<br>1:1000 (TH)	711-605-152<br>(Jackson ImmunoResearch)	IHC
**Anti-mouse**	Cy3-donkey anti-mouse IgG	1:500	715-165-150<br>(Jackson ImmunoResearch)	IHC
